# Increased HIF-1*α* in Knee Osteoarthritis Aggravate Synovial Fibrosis via Fibroblast-Like Synoviocyte Pyroptosis

**DOI:** 10.1155/2019/6326517

**Published:** 2019-01-10

**Authors:** Li Zhang, Li Zhang, Zhengquan Huang, Runlin Xing, Xiaochen Li, Songjiang Yin, Jun Mao, Nongshan Zhang, Wei Mei, Liang Ding, Peimin Wang

**Affiliations:** Department of Orthopedics, The Affiliated Hospital of Nanjing University of Chinese Medicine, Nanjing, China

## Abstract

Fibroblast-like synoviocytes (FLSs) are the main effector cells of knee osteoarthritis (KOA) synovial fibrosis. Our last report showed that NLRP1 and NLRP3 inflammasomes may mediate LPS/ATP-induced FLSs pyroptosis in KOA. In the present study, we found an elevated hypoxia-inducible factor-1*α* (HIF-1*α*) level in the synovial tissue of KOA model rats, and inhibiting the increase of HIF-1*α* could improve synovial fibrosis in rats. Subsequently, we established LPS/ATP-induced model in FLSs mimicking the inflammatory environment of KOA. FLSs transfected with siRNA HIF-1*α* showed a reduced cell death; meanwhile, the relative expression of pyroptosis-related proteins was also downregulated. Additionally, FLSs transfected with or without siRNA GSDMD were exposed to hypoxia. GSDMD silencing can significantly reduce both gene and protein levels of fibrogenic markers transforming growth factor-*β* (TGF-*β*), procollagen-lysine, 2-oxoglutarate 5-dioxygenase2 (PLOD2), collagen type I *α*1 chain (COL1A1), and tissue inhibitor of metalloproteinases 1 (TIMP1). Taken together, our findings indicate that increased HIF-1*α* is highly involved in the KOA synovial fibrosis. Moreover, elevated HIF-1*α* may aggravate synovial fibrosis via FLS pyroptosis.

## 1. Introduction

Knee osteoarthritis (KOA) is a degenerative joint disorder that affects all tissues in the knees characterized by the progressive destruction of articular cartilage and surrounding tissues especially the synovial membrane, causing pain, stiffness, and chronic disability [1]. Among these symptoms, stiffness and pain are highly correlated with synovial fibrosis and fibroblast-like synoviocytes (FLSs) have been proven to be the main effector cells [2]. In KOA, excessive extracellular matrix (ECM) deposition in the presence of inflammation and tissue damage leads to synovial fibrosis. Accumulating evidence has demonstrated the critical role of transforming growth factor-*β* (TGF-*β*) in fibrotic response [3, 4]. Besides, genes procollagen-lysine, 2-oxoglutarate 5-dioxygenase2 (PLOD2) and collagen type I *α*1 chain (COL1A1) and tissue inhibitor of metalloproteinases 1 (TIMP1) are shown upregulating in osteoarthritis-related fibrosis; they are usually considered to be fibrogenic markers [5].

With the development of research, KOA has been widely accepted as an immune-related disease. Thus, inflammation and oxidative stress are frequently discussed in the onset and progression of KOA [1].

Recently, a new form of programmed inflammatory cell death has been described as pyroptosis. This caspase-1-dependent cell death is mostly found in monocytes, macrophages, and dendritic cells [6]. In the canonical process of pyroptosis, activated nod-like receptor protein 3 (NLRP3) forms an inflammasome comprised of apoptosis-associated speck-like protein (ASC) and the serine protease caspase-1 [7]. NLRP3 inflammasome governs the cleavage and activation of caspase-1; subsequently, the activated caspase-1 will further generate proinflammatory cytokines IL-1*β* and IL-18 by the cleavage of their precursor and induces pyroptosis through the cleavage of gasdermin D (GSDMD) [8].

In our last study [9], the correlation between the NLRP inflammasomes and FLS pyroptosis was investigated in vivo and in vitro. We have revealed that NLRP1 and NLRP3 inflammasomes mediate LPS/ATP-induced pyroptosis in KOA. However, the significance of FLS pyroptosis in KOA still needs to be further explored. Increasing evidence has shown that cell pyroptosis in different tissues such as the liver, renal, and airway participates in chronic aseptic inflammation and can be related to tissue fibrosis [10–12]. Would this also be applied to explain the pathological mechanism of synovial fibrosis in KOA?

The knee joints consume oxygen and nutrients during movement; thus, it is very important to study the homeostasis of the cellular environment from the perspective of oxidative stress [13]. Hypoxia-inducible factor-1*α* (HIF-1*α*) is a transcriptional factor and key regulator of the cellular response to hypoxia. Clinical studies have shown that HIF-1*α* levels in the serum, synovial fluid, and articular cartilage of KOA patients are associated with progressive joint damage [14]. It may serve as an alternative biomarker for the progression and prognosis of KOA. Previous studies have demonstrated that HIF-1*α* is associated with the upregulation of gene expression encoding proinflammatory cytokines and growth factors to activate fibroblasts and mediate fibrosis [15]. Unfortunately, the specific molecular mechanism in KOA synovial fibrosis involving HIF-1*α* is still unknown.

In summary, synovial fibrosis in KOA seems to be a combined result of inflammation and oxidative stress. FLS pyroptosis may play a crucial role in the entire pathogenesis of KOA. This programmed inflammatory cell death may be regulated by HIF-1*α* and associated with synovial fibrosis. In the present study, we investigated the correlation between HIF-1*α*, FLS pyroptosis and synovial fibrosis in vitro cells, and KOA model rats via molecular biology and histochemical methods. Our hypothesis is that elevated HIF-1*α* in KOA may aggravate synovial fibrosis via FLS pyroptosis. Ameliorating hypoxia in the synovial tissue or inhibition of FLS pyroptosis may exert a protective effect.

## 2. Materials and Methods

### 2.1. In Vivo Animal Experimental Design

Eighteen 3-month-old SD female rats, weight ranging from 280 g to 320 g (provided by Beijing Vital River Laboratory Animal Technology Co. Ltd.), were used. Animals were maintained in a specific pathogen-free laminar-flow housing apparatus under controlled temperature, humidity, and 12 h light/dark regimen. All animal protocols were approved by the Animal Care and Use Committee of the Nanjing University of Chinese Medicine. All experiments were conducted in accordance with the National Institutes of Health Guidelines for the Care and Use of Laboratory Animals.

Rats were randomly assigned to three groups: Normal (*n* = 6), KOA (*n* = 6), and KOA+Digoxin (*n* = 6). KOA was induced by monosodium iodoacetate (MIA) as described previously. Briefly, on day 1, KOA group rats were anesthetized with Nembutal and given intra-articular injection of 1 mg of MIA (Sigma, St. Louis, MO, USA) in 50 *μ*l physiologic saline through the infrapatellar ligament of the bilateral knee joint while Normal group rats were treated with 50 *μ*l sterilized physiologic saline. From day 14, the KOA+Digoxin group was intraperitoneally injected with HIF-1*α* inhibitor digoxin (Chengdu Herb Purity Co. Ltd., Chengdu, China) at a dose of 100 *μ*g/kg, in 50 *μ*l sterilized physiologic saline, once a day for 2 weeks, while the other two groups were treated with 50 *μ*l sterilized physiologic saline. At day 28, all rats were sacrificed to harvest the synovial tissue.

### 2.2. Measurement of the Right Knee Joint Diameter

The transverse diameters of the right knees were measured at day 1, day 7, day 14, day 21, and day 28, with a slide caliper (Mitutoyo, Kanagawa, Japan) to measure the horizontal distance between the left and right highest points of the knee joints flexed at 90°, respectively. Each knee was measured three times, and the mean value was calculated.

### 2.3. Hematoxylin and Eosin Staining

Synovial tissues were fixed in 10% neutral formalin, soaked in EDTA, embedded in paraffin, and cut into slices, for routine HE staining.

### 2.4. Sirius Red Staining

All procedures were carried out according to the instructions of Picro Sirius Red Stain kit (Abcam, Cambridge, UK). Briefly, the tissue sections were dewaxed, dipped into water, stained with 1 g/l Picric Acid-Sirius Red at 37°C for 1 h, and then washed with water. The sections were mounted and viewed under a Leica DMI3000B microscope (Leica, Germany), with the use of bright field.

### 2.5. Isolation and Culture of Primary Rat KOA-FLSs

In brief, synovial tissues were washed for 2-3 times with phosphate-buffered saline (PBS) then minced into pieces of 2-3 mm^2^, digested in 0.1% collagenase type II (Sigma, St. Louis, MO, USA) for 30 min. Following cell dissociation, the samples were filtered through a cell strainer. After dissociation, fibroblasts were pelleted by centrifugation at 1,500 rpm for 4 min and cultured in DMEM supplemented with 10% fetal calf serum (FCS; Gibco, Thermo Fisher Scientific, Waltham, MA, USA) and antibiotics (100 U/ml penicillin, 100 *μ*g/ml streptomycin; Invitrogen, CA, USA). Cells were identified as our previous studies [16]. Passages 3-6 of the synovial fibroblasts were used for the experiments.

### 2.6. Small Interfering RNA Preparation and Transfection

To inhibit the HIF-1*α* and GSDMD expression in the FLS, commercially available HIF-1*α*, GSDMD, and vehicle control small interfering RNA (Invitrogen, CA, USA) were used. FLSs were transfected with siRNAs by using Lipofectamine 2000 (Invitrogen, CA, USA) according to manufacturer's instructions. siRNA was diluted in transfection reagent and culture medium, and the cells were incubated with 20 pmol siRNA for 6 h.

### 2.7. FLS Treatment

To induce cell pyroptosis, FLSs transfected with HIF-1*α* siRNA (HIF-1*α* siRNA group) or vehicle scramble siRNA (Vehicle group) were stimulated with LPS (1 *μ*g/ml) in DMEM for 6 h and then challenged with ATP (3 mM) for 1 h. The FLSs exposed to DMEM with same volume of PBS served as controls (Normal group).

To evaluate the relationship between pyroptosis and fibrosis, FLSs transfected with GSDMD siRNA (GSDMD siRNA group) or vehicle scramble siRNA (Vehicle group) were exposed to hypoxia (cultured in 1% O_2_, 24 h). The Normal group FLSs cultured at 37°C in a humidified 95% air and 5% CO_2_ atmosphere served as controls (Normal group).

### 2.8. Real-Time PCR

RNA was isolated from synovial tissue and FLSs with Trizol (Invitrogen, CA, USA), respectively. The reverse transcription was performed by using a first strand cDNA synthesis kit (Takara, Otsu, Japan) according to manufacturer's instructions. qPCR was performed using Premix Ex Taq SYBR-Green PCR (Takara) according to manufacturer's instructions on an ABI PRISM 7300 (Applied Biosystems, Foster City, CA, USA).

Primer was designed and synthesized by Shanghai Biotechnology Service Company in accordance with the gene sequence in GenBank gene sequence design, together with Oligo v6.6. Sequences for primers were as follows: caspase-1 forward, 5′-GACCGAGTGGTTCCCTCAAG-3′, and reverse, 5′-GACGTGTACGAGTGGGTGTT-3′; ASC forward, 5′-GACAGTACCAGGCAGTTCGT-3′, and reverse, 5′-AGTAGGGCTGTGTTTGCCTC-3′; NLRP3 forward, 5′-CTCACCTCACACTCCTGCTG-3′, and reverse, 5′-AGAACCTCACAGAGCGTCAC-3′; GSDMD forward, 5′-TCATGGTTCTGGAAACCCCG-3′, and reverse, 5′-CCAGACACTGGTTCTGGAGC-3′; TGF-*β* forward, 5′-GACTCTCCACCTGCAAGACC-3′, and reverse, 5′-GGACTGGCGAGCCTTAGTTT-3′; COL1A1 forward, 5′-GTACATCAGCCCAAACCCCA-3′, and reverse, 5′-CAGGATCGGAACCTTCGCTT-3′; PLOD2 forward, 5′-ATGCTCGAGACATGGGTGTG-3′, and reverse 5′-TTTTCCTTCCAATCCACGGG-3′; TIMP1 forward, 5′-CAGCTTTCTGCAACTCGGAC-3′, and reverse, 5′-CAGCGTCGAATCCTTTGAGC-3′; and GAPDH forward, 5′-TTCACCACCATGGAGAAGGC-3′, and reverse, 5′-CTCGTGGTTCACACCCATCA-3′. The mRNA level of individual genes was normalized to GAPDH and calculated by 2^−ΔΔCt^ data analysis method.

### 2.9. Western Blotting Analysis

Synovial tissues and FLS were mixed with RIPA lysate and grinded for 10-15 min, respectively. Samples were agitated on ice for 30 min and the supernatant was collected. The protein levels were quantified with a BCA protein assay kit (Roche, Basel, Switzerland). Then the protein samples were electrophoresed in SD-PAGE to separate protein bands. Proteins were transferred from gel onto PVDF membrane, blocked with 5% nonfat dry milk for 2 h. The membrane was incubated with the first antibody (1 : 1000) for overnight at 4°C, and then with the second antibody for 2 hours. Later bands were visualized by exposure to ECL method and the overall gray value of protein bands (average gray value area) was quantified, GAPDH as internal marker, namely, target protein gray value/internal reference overall gray value.

### 2.10. ELISA

IL-1*β* and IL-18 levels in the culture media were determined using a commercially available rat IL-1*β*, IL-18 enzyme-linked immunosorbent assay (ELISA) kit (Shanghai Lengton Bioscience Co., Shanghai, China) according to manufacturer's instructions.

### 2.11. Flow Cytometry

After treatment, the cells were double stained with Annexin V-fluorescein isothiocyanate and propidium iodide (Annexin V: FITC apoptosis detection kit; BD Biosciences, Santa Cruz, CA, USA) according to the instructions. Quantification was then performed by flow cytometry (Beckman Coulter, Miami, FL, USA).

### 2.12. Pimonidazole Staining and Immunofluorescence

To investigate synovial tissue hypoxia, rats were injected with Hypoxyprobe™-1 (pimonidazole HCl) (Hypoxyprobe™-1 Plus Kit, Burlington, MA, USA) at a dosage of 60 mg/kg for 45 min prior to sacrifice. Subsequent immunofluorescence staining followed the kit instructions. The image was observed by inverted fluorescence microscope (Leica DMI3000B, Germany).

To observe intracellular hypoxia, treated FLSs were fixed with 4% paraformaldehyde in PBS for 15 min and then blocked with 5% BSA for 60 min. Subsequent incubation with primary antibodies and secondary antibodies followed the kit instructions; cells were visualized under a confocal scanning microscope (Zeiss LSM 700).

### 2.13. Statistical Analysis

Statistical analysis was performed using GraphPad Prism 6.0 Software (San Diego, CA, USA). Data are presented as mean ± standard deviation. Group comparisons were assessed with the one-way ANOVA or Student's *t*-test or two-way ANOVA with Bonferroni's post hoc test for comparison of multiple columns. A value of *P* < 0.05 (two-tailed) was considered as statistically significant.

## 3. Results

### 3.1. The Synovial Tissue of KOA Rats Was in a State of Aggravated Hypoxia

**Figure 1:**
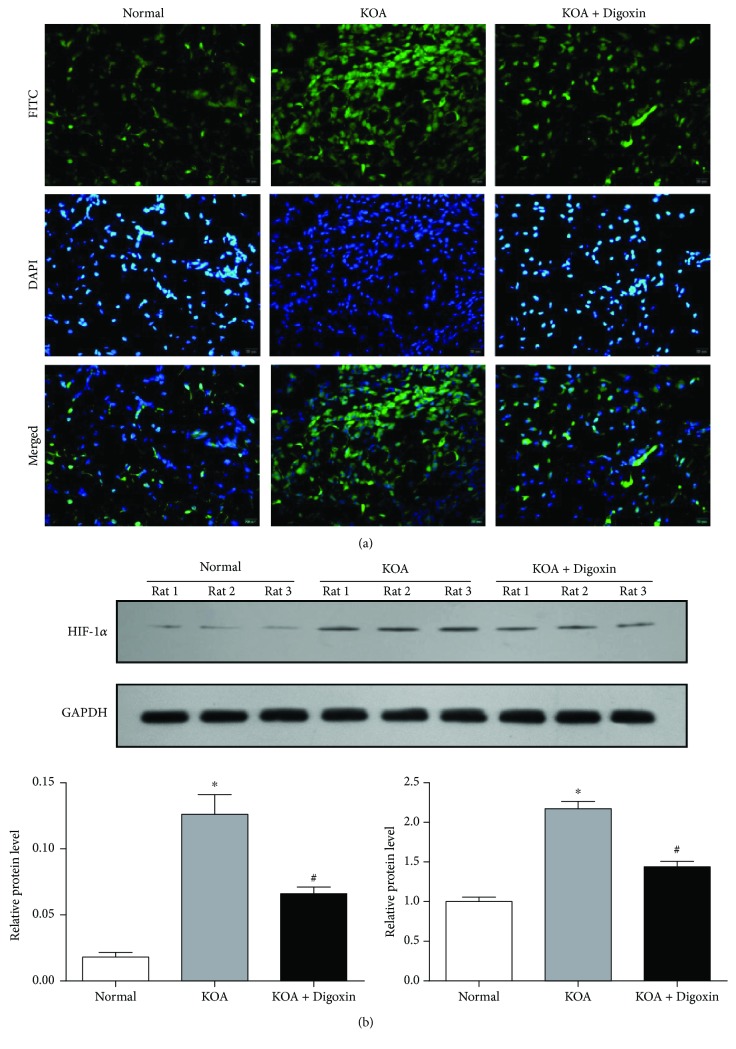
The synovial tissue of KOA rats was in a state of aggravated hypoxia. (a) Representative synovial tissues stained with pimonidazole, 400x, scale bar = 20 *μ*m. (b) Relative gene and protein expressions of HIF-1*α* in synovial tissues of rats in each group. Data were expressed as the mean ± SD. ^∗^*P* < 0.05 vs. the Normal group; ^#^*P* < 0.05 vs. the KOA group.

The synovial tissue of MIA-induced KOA model rats shows an aggravated hypoxia compared with the Normal group observed by pimonidazole staining (Figure 1(a)) and HIF-1*α* inhibitor digoxin (100 *μ*g/kg) could be able to contain this situation. In addition, we quantitatively analyzed both gene and protein expressions (Figure 1(b)) of HIF-1*α* in synovial tissues of rats in each group. The KOA group resulted in a significant upregulation compared with the Normal group (*P* < 0.05). This upregulation was significantly inhibited in the KOA+Digoxin group (*P* < 0.05).

### 3.2. HIF-1*α* Inhibitor Attenuated Synovial Fibrosis in Rats

**Figure 2:**
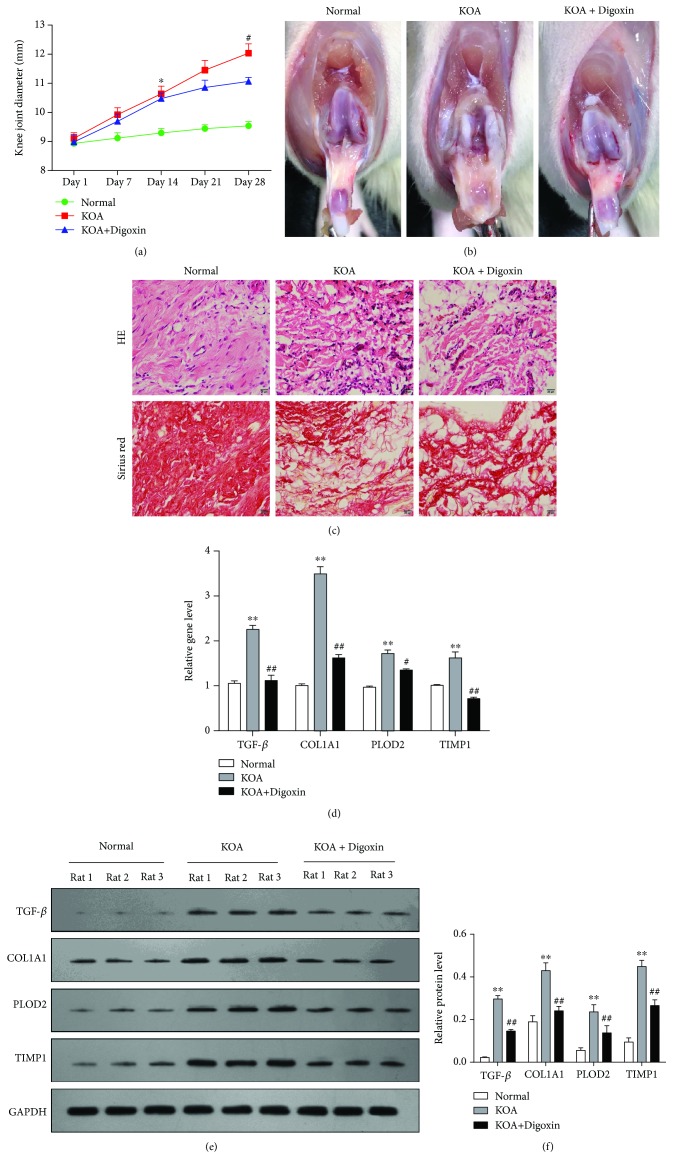
HIF-1*α* inhibitor attenuated synovial fibrosis in rats. (a) The severity of synovial fibrosis evaluated by the transverse diameters of the right knees. ^∗^*P* < 0.05 vs. the Normal group; ^#^*P* < 0.01 vs. the KOA+Digoxin group. (b) Anatomical changes of each group. (c) Representative synovial tissues of each group stained with HE and Sirius Red staining, 200x, scale bar = 20 *μ*m. Disorderly arranged synovial lining cells, inflammatory cell infiltration, and increased collagen deposition could be observed in the KOA group. (d) Relative gene expression of TGF-*β*, COL1A1, PLOD2, and TIMP1 in synovial tissues of rats in each group. ^∗∗^*P* < 0.01 vs. the Normal group; ^##^*P* < 0.01 vs. the KOA group. (e) Typical protein bands for each group. (f) The same trend with gene level can also be observed in the relative protein level.

To quantify the degree of knee swelling in rats, transverse diameters of the right knees were measured (Figure 2(a)). On day 14, the knee joint diameter of the KOA group was significantly larger than that of the Normal group (*P* < 0.05), and on day 28, the knee joint diameter of the KOA+Digoxin group was significantly smaller than that of the KOA group (*P* < 0.05). To evaluate synovial fibrosis in rats, anatomical characteristics and pathological sections of the synovial tissue (Figures 2(b) and 2(c)) were observed. In HE staining, digoxin treatment showed an orderly arranged synovial lining cells, loose connective tissue, and less inflammatory cell infiltration, compared with the KOA rat. Besides, the KOA group showed markedly increased collagen deposition while this change was relatively lessened in the KOA+Digoxin group observed under Sirius Red staining. In addition, the gene expressions of TGF-*β*, COL1A1, PLOD2, and TIMP1 were measured by real-time PCR (Figure 2(b)). There was a significant increase in the expression of these fibrosis-related genes in the KOA group compared with the Normal group (*P* < 0.01); digoxin treatment could attenuate the upregulation of these genes. The same trend can also be observed with Western blotting (Figures 2(c) and 2(d)) after gene translation into proteins (*P* < 0.01).

### 3.3. HIF-1*α* siRNA Attenuates the LPS+ATP-Induced Cell Pyroptosis in FLS

**Figure 3:**
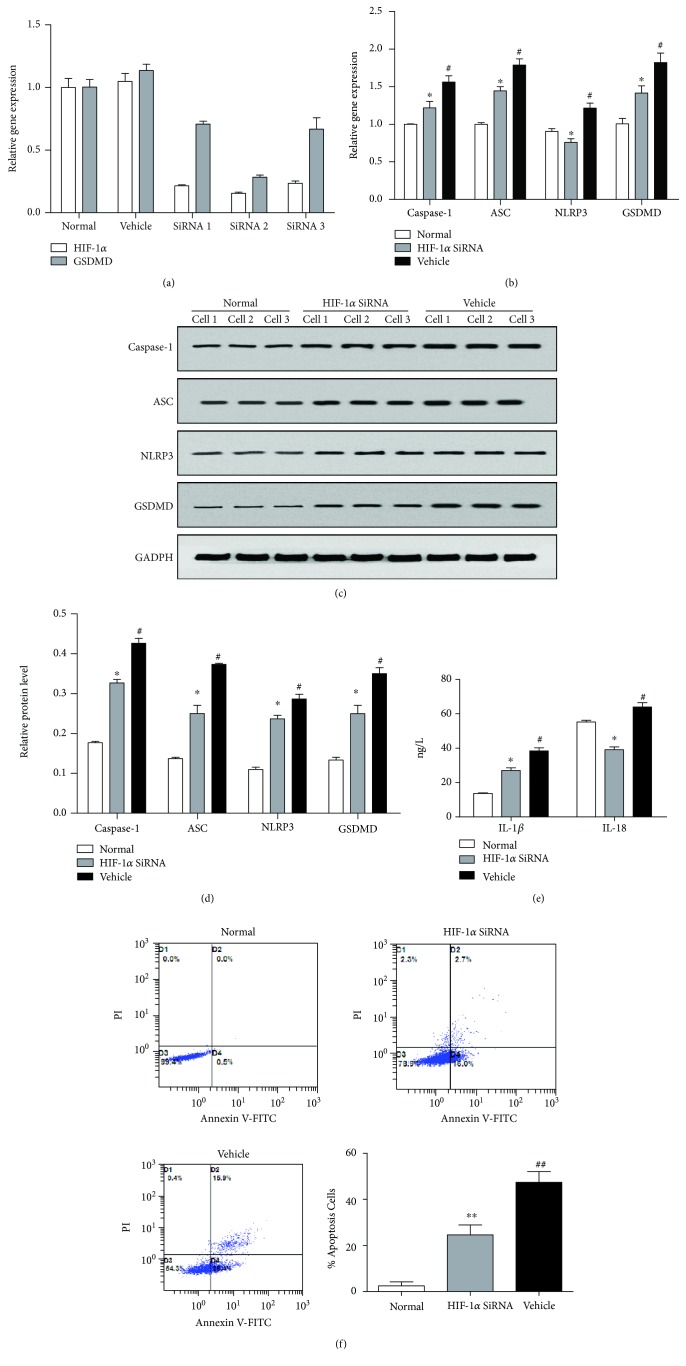
HIF-1*α* siRNA attenuates the LPS+ATP-induced cell pyroptosis in FLS. (a) The silencing effect of the HIF-1*α* siRNA and GSDMD siRNA was both confirmed by PCR. (b) mRNA expression of NLRP3 inflammasome, its components (caspase-1, ASC), and its downstream (GSDMD) were downregulated after the transfection of HIF-1*α* siRNA compared with the Vehicle group. (c) Typical protein bands for each group. (d) The same trend with gene level could also be observed in the relative protein level. ^∗^*P* < 0.05 vs. the Vehicle group; ^#^*P* < 0.05 vs. the Normal group. (e) The HIF-1*α* siRNA group has shown a significant downregulation of IL-1*β* and IL-18 compared with the Vehicle group. ^∗^*P* < 0.05 vs. the Vehicle group; ^#^*P* < 0.05 vs. the Normal group. (f) The cell apoptosis rate in the Vehicle group was significantly higher than that in the Normal group (*P* < 0.01), and the cell apoptosis rate in the HIF-1*α* siRNA group was significantly lower than that in the Vehicle group (*P* < 0.01).

The silencing effect of the HIF-1*α* siRNA and GSDMD siRNA was confirmed by PCR. After LPS+ATP treatment, the protein and relative mRNA expression (Figures 3(b)–3(d)) of caspase-1, ASC, NLRP3, and GSDMD in the Vehicle group were higher than those in the Normal group (*P* < 0.05); meanwhile, HIF-1*α* siRNA group has shown a significant decrease of these key pyroptosis-related substances compared with the Vehicle group (*P* < 0.05). Levels of proinflammatory factors IL-1*β* and IL-18 (Figure 3(e)) released by FLS pyroptosis in culture supernatant were determined by ELISA. The HIF-1*α* siRNA group has shown a significant downregulation of IL-1*β* and IL-18 compared with the Vehicle group (*P* < 0.05). In addition, cell apoptosis was detected by flow cytometry (Figures 3(f)). The cell apoptosis rate in the Vehicle group was significantly higher than that in the Normal group (*P* < 0.01), and the cell apoptosis rate in the HIF-1*α* siRNA group was significantly lower than that in the Vehicle group (*P* < 0.01).

### 3.4. Inhibition of FLS Pyroptosis May Alleviate Fibrosis

**Figure 4:**
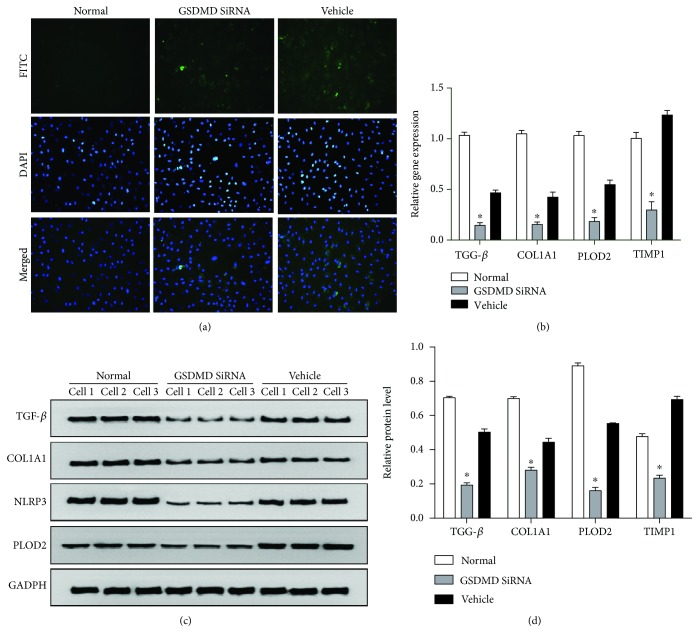
Inhibition of FLS pyroptosis may alleviate fibrosis. (a) Representative synovial tissues stained with pimonidazole, 400x, scale bar = 20 *μ*m. The Vehicle group and the GSDMD siRNA group showed similar intracellular hypoxia. (b) Relative gene expressions of TGF-*β*, COL1A1, PLOD2, and TIMP1 in the GSDMD siRNA group were downregulated compared with those in the Vehicle group (*P* < 0.05). (c) Typical protein bands for each group. (d) Protein levels of TGF-*β*, COL1A1, PLOD2, and TIMP1 in the GSDMD siRNA group were downregulated compared with those in the Vehicle group (*P* < 0.05).

After the Vehicle and GSDMD siRNA groups were exposed to hypoxia (1% O_2_) for 24 h, pimonidazole staining was performed to confirm intracellular hypoxia (Figure 4(a)). The Vehicle group and the GSDMD siRNA group showed similar intracellular hypoxia. Then PCR (Figure 4(b)) and Western blotting (Figures 4(c) and 4(d)) were used to evaluate fibrosis at the level of gene and protein expression. Both mRNA and protein expressions of TGF-*β*, COL1A1, PLOD2, and TIMP1 were markedly downregulated in GSDMD siRNA-transfected FLS compared with Vehicle (*P* < 0.05).

## 4. Discussions

In the present study, the relationship between HIF-1*α* and synovial fibrosis was investigated in KOA rats. The inhibition of HIF-1*α* in KOA downregulated the level of synovial fibrosis-related factors TGF-*β*, COL1A1, PLOD2, and TIMP1. In subsequent experiments in vitro, we proved that HIF-1*α* siRNA may lead to a significant reduction of cell pyroptosis-related factors caspase-1, ASC, NLRP3, GSDMD, IL-1*β*, and IL-18, both mRNA and protein expressions, compared with those with normal FLSs after the treatment of LPS+ATP. In addition, when FLSs were exposed to hypoxia, GSDMD siRNA may reduce the gene and protein levels of these aforementioned synovial fibrosis-related factors. These findings indicated that increased HIF-1*α* in KOA may aggravate synovial fibrosis via FLS pyroptosis.

Hypoxia and inflammation persistently exist in the pathological progress of KOA. It is well established that when tissue oxygen demand exceeds supply, a cascade of intracellular events is activated increasing the expression of HIF-1*α*. Previous studies have demonstrated that increased HIF-1*α* could cause chondrocyte apoptosis through the overactivation of endoplasmic reticulum stress [17]. As a transcription factor, HIF-1*α* regulates the expression of genes encoding proteins involved in angiogenic growth factors, such as vascular endothelial growth factor (VEGF) and TGF-*β*, in rheumatoid arthritis [18].

In addition, Gupta et al. found that HIF-1*α* can induce NLRP3 inflammasome complex during hypoxic conditions [19]. NLRP3 is one of the most well-known inflammasomes, which can activate caspase-1 and induce pyroptosis. This canonical pathway may require a two-step mechanism. At the first stage, proinflammatory mediators pro-IL-1*β* and pro-IL-18, NLRP3, and caspase family members are transcriptionally generated. This stage is called the preparatory phase and has been proven to be highly correlated with the NF-*κ*B signaling pathway. The second stage is activation, when the NLRP3 inflammasomes are assembled and caspase-1 is activated. Activated caspase-1 further matures pro-IL-1*β* and pro-IL-18 and induces pyroptosis partially through the cleavage of GSDMD [20]. Unlike the NLRP3/caspase-1 canonical pathway pyroptosis, caspase-11 can be activated by direct sensing of intracellular LPS and promotes pyroptosis through GSDMD cleavage [21]. It seems that the cleavage of GSDMD is required and sufficient to drive pyroptosis. Shi et al. confirmed this core role of GSDMD cleavage in cell pyroptosis through CRISPR-Cas9 [22]. Pyroptosis causes rapid plasma membrane rupture, resulting in the release of intracellular proinflammatory mediators IL-1*β*, IL-18, and HMGB1 [23].

Among the various proinflammatory cytokines released from pyroptosis, IL-1*β* has been the one most extensively studied in fibrotic diseases, such as liver, lung, and kidney fibrosis. In vitro studies have shown that IL-1*β* promotes the proliferation and transdifferentiation of HSCs with a substantial increase in levels of their fibrogenic markers, including metalloproteinases (MMPs), TIMP1, COL1A1, and TGF-*β* [24]. TGF-*β* is considered to be the master regulator of fibrosis because it activates FLSs, promotes expression of ECM genes, and inhibits ECM degradation. Gene expressions of PLOD2, COL1A1, and TIMP1 were upregulated both in OA fibroblasts stimulated with TGF-*β* and in mice with TGF-*β*-induced fibrosis [25]. Besides, TGF-*β* is associated with various signaling pathways, such as Smad and WNT, or microRNA to regulate chronic inflammation and fibrosis in joint tissues [26–28]. Thus, it is generally considered that TGF-*β* is a crucial mediator linking inflammation to fibrosis.

As mentioned above, pyroptosis induced by the NLRP3 inflammasome is receiving increasing attention in the recent researches since it participates in various diseases. Multiple mechanisms have been proposed to activate NLRP3, including bacterial, K+ efflux, lysosomal destabilization, mitochondrial damage, production of reactive oxygen species, Ca2+ influx, and cell swelling. The study of pyroptosis is initially concentrated on macrophages infected by bacteria and virus. But increasing evidence has shown that cell pyroptosis may occur in different tissues participating in chronic aseptic inflammation and related to tissue fibrosis. For instance, microglia pyroptosis can induce neurogenic inflammation and fibrosis [29], hepatic stellate cell pyroptosis causes chronic hepatitis and liver fibrosis [10], and macrophage pyroptosis in the kidney leads to renal inflammation and fibrosis [30].

In the present paper, we premise that increased HIF-1*α* in KOA may aggravate synovial fibrosis via FLS pyroptosis. This hypothesis was confirmed in subsequent studies. The synovial tissue of KOA model rats showed a significant increase in mRNA and protein expression of HIF-1*α*. Inhibiting the increase of HIF-1*α* can reduce the level of fibrogenic markers TGF-*β*, COL1A1, PLOD2, and TIMP1. This in vivo experiment demonstrated that hypoxia was highly correlated with KOA synovial fibrosis. In the subsequent in vitro experiments, HIF-1*α* silencing can significantly reduce the FLS death induced by LPS+ATP; meanwhile, both gene and protein levels of caspase-1, ASC, NLRP3, GSDMD, IL-1*β*, and IL-18 were also downregulated. This may indicate that pyroptosis was involved in KOA and can be induced by elevated HIF-1*α* because LPS stimuli can mimic inflammatory environments similar to KOA and are often used to induce arthritis models. At last, FLSs transfected with or without siRNA GSDMD were exposed to hypoxia. GSDMD silencing can significantly reduce both gene and protein levels of fibrogenic markers TGF-*β*, COL1A1, PLOD2, and TIMP1. This data suggests that FLS pyroptosis may exacerbate fibrosis in hypoxia.

## 5. Conclusion

Based on the data obtained from rats and in vitro cells, we concluded that increased HIF-1*α* is highly involved in the KOA synovial fibrosis. Moreover, elevated HIF-1*α* may aggravate synovial fibrosis via FLS pyroptosis. Further study is required to determine the signal pathway involved in HIF-1*α*/NLRP3 inflammasome activation/pyroptosis. Its mechanism will provide new ideas and means for understanding and treating KOA-related synovial fibrosis.

## Data Availability

The data used to support the findings of this study are available from the corresponding author upon request.

## Abbreviations

KOA: Knee osteoarthritis
FLS: Fibroblast-like synoviocytes
NLRP: Nod-like receptor protein
ASC: Apoptosis-associated speck-like protein with a caspase recruitment domain
GSDMD: Gasdermin D
LPS: Lipopolysaccharide
ATP: Adenosine triphosphate
siRNAs: Small interfering RNAs
HIF-1*α*: Hypoxia-inducible factor-1*α*
TGF-*β*: Transforming growth factor-*β*
PLOD2: Procollagen-lysine, 2-oxoglutarate 5-dioxygenase2
COL1A1: Collagen type I *α*1 chain
TIMP1: Tissue inhibitor of metalloproteinases 1.
